# A novel suicide gene therapy for the treatment of p16^Ink4a^-overexpressing tumors

**DOI:** 10.18632/oncotarget.23752

**Published:** 2017-12-28

**Authors:** Jaskaren Kohli, Judith Campisi, Marco Demaria

**Affiliations:** ^1^ European Research Institute for the Biology of Aging, University Medical Center Groningen, University of Groningen, Groningen, Netherlands; ^2^ Buck Institute for Research on Aging, Novato, CA, USA; ^3^ Lawrence Berkeley National Laboratory, Life Sciences Division, Berkeley, CA, USA

**Keywords:** p16^Ink4a^, sarcoma, cell cycle, cellular senescence, p53

## Abstract

p16^Ink4a^ is a potent cell cycle inhibitor engaged to support cell cycle arrest during cellular senescence. However, in tumors carrying mutations in key downstream effectors, p16^Ink4a^ is highly expressed but fails to block cell proliferation. p16^Ink4a^-overexpressing tumor cells are highly aggressive and no targeted interventions are available. To study the effect of specific therapies, we generated murine sarcomas by overexpressing RAS oncogene and disrupting p53 activity. We observed that p16^Ink4a^-overxpressing murine sarcoma cells were resistant to ABT-263 and ABT-737, anti-cancer small molecules previously shown to eliminate p16^Ink4a+^ senescent cells. We then generated sarcoma cells carrying a suicide and reporter gene, called 3MR, under the regulation of the full p16^Ink4a^ promoter. Activation of the suicide efficiently killed p16^Ink4a^-overxpressing sarcoma cells *in vitro* and *in vivo*.

These data suggest that suicide gene therapy could represent an important therapeutic approach for the treatment of highly aggressive p16^Ink4a+^ cancers.

## INTRODUCTION

p16^Ink4a^ is the principal member of the Ink4 family of Cyclin-Dependent Kinase (CDK) inhibitors, which arrests cell cycle progression by inhibiting the S phase [1]. Specifically, p16^Ink4a^ blocks CDK4/6 complexes from interacting with cyclinD1 thus maintaining the retinoblastoma protein (RB) in a hypo-phosphorylated and active state [2]. Active RB associates with E2F1, an essential transcriptional factor for the induction of genes associated to G1-S transition, and inhibits its translocation to the nucleus [2]. Cellular senescence, a tumor suppressive mechanism defined as irreversible growth arrest and induced by accumulation of DNA damage, is often associated to induction of p16^Ink4a^ [3]. Consequently, p16^Ink4a^ is considered a strong tumor suppressor, and mice lacking both copies of p16^Ink4a^ are extremely susceptible to tumorigenesis [4]. Loss-of-function mutations affecting p16^Ink4a^ are a common mark of various human tumors, and considered an essential step towards tumor progression [5]. However, in the presence of mutations affecting RB or CDK4/6, p16^Ink4a^ activity is not sufficient to arrest cell cycle progression [6]. Moreover, p16^Ink4a^ overexpression has been observed at the invasive front of endometrial, colorectal and basal cell carcinoma and correlated with high aggressiveness [7]. Thus, under these conditions targeting p16^Ink4a^-overexpressing cells could be a potent anti-cancer intervention. Here, we observe that p16^Ink4a^-overexpressing sarcoma cells are resistant to small molecules shown to be toxic against p16^Ink4a +^-senescent cells, but are sensitive to a novel suicide gene therapy regulated by the full *p16*^*Ink4a*^ promoter.

## RESULTS

### p16^Ink4a^-overexpressing sarcoma cells are resistant to senolytic agents

**Figure 1:**
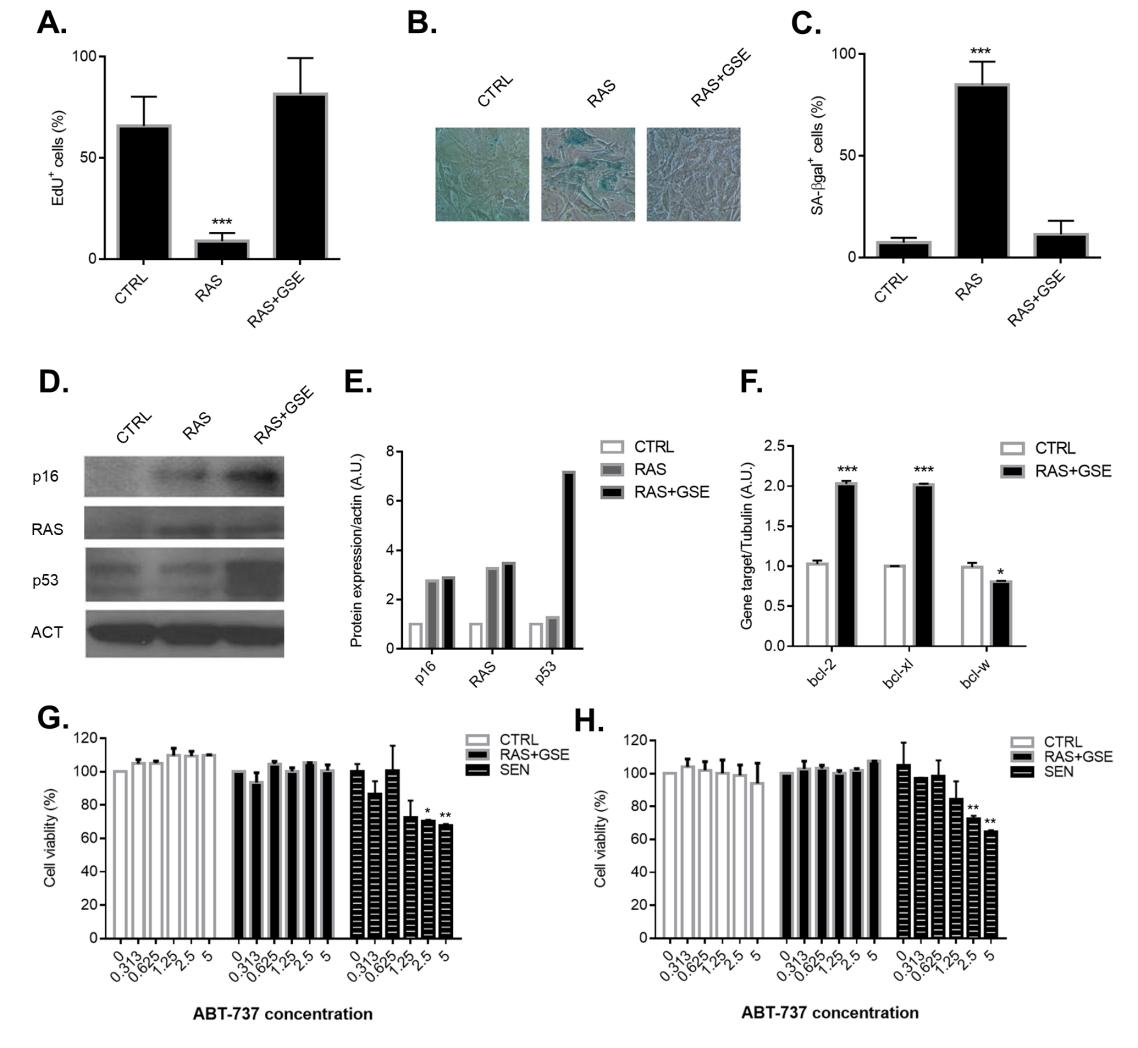
Characterization of p16^ink4a^-overexpressing sarcomas and treatment with senolytic drugs Primary Mouse Embryonic Fibroblasts (MEFs) were transduced with lentivirus containing vector control (CTRL), RASVal12 (RAS) or RASVal12 and p53-GSE (RAS+GSE). **A.**-**C.** 5 days after selection, cells were either incubated for 24 hrs with EdU then fixed and stained (A) or fixed and stained for SA-β-gal (B-C). In A and C is shown the percentage of positive cells (>100 cells scored). In B, a representative image of SA-β-gal staining. *N* = 3 independent experiments. **D.-E.** p16, RAS and p53 protein levels were measured by immunoblotting using whole cell extracts from CTRL, RAS or RAS+GSE MEFs. Actin was used as a loading control. (D) shows the blot, while graph in E represents the quantification obtained using ImageJ. **F.** Quantitative real-time PCR (qRT-PCR) analysis of RNA isolated from CTRL or RAS+GSE MEFs. RNA was analyzed for mRNAs encoding bcl-2, bcl-xl and bcl-w relative to tubulin (to control for cDNA quantity). *N* = 3 technical replicates. A.U.=arbitrary units. **G.**-**H.** CTRL, RAS+GSE or irradiated (SEN) MEFs were treated with the indicated concentrations of ABT-263 (F) or ABT-737 (G) 3 times for 24 hours. Cells were evaluated for viability using a MTS assay. The graph reports the absorbance of each sample as a percentage of control. *N* = 2 independent experiments with 3 technical replicates. *p<=0.05, **<=0.01, ***p<=0.001.

Cellular transformation requires the activation of proto-oncogenes and/or inhibition of oncosuppressors - more often a combination of the two. Activating mutations in the RAS family of proto-oncogenes are a common event during tumorigenesis, and detected in 20-30% of all human cancers [8]. However, the sole overexpression of a mutated form of RAS (RASv12) in primary cells is associated to induction of irreversible growth arrest, a phenomenon also known as oncogene-induced senescence [2]. Indeed, overexpression of a mutated form of RAS (RASv12) induced loss of proliferation and activation of the senescence-associated β galactosidase enzyme (SA-βgal) (Figure 1A-1C) in Mouse Embryonic Fibroblasts (MEFs). Activation of a senescence program was associated with upregulation of p16^Ink4a^ levels (Figure 1D). Another common lesion in tumors is loss-of-function mutations in the oncosuppressor p53. Combination of RAS overexpression with p53 inactivation, obtained through the use of a Genetic Suppressor Element (GSE) which interferes with p53 tetramerization [9], was sufficient to overcome both cycle arrest and activation of SA-βgal (Figure 1A-1C). However, despite the by-pass of the senescence program, cells overexpressing RAS and with inactive p53 induced high level of p16^Ink4a^ (Figure 1D-1E). We then hypothesized that treatment with compounds shown to selectively eliminate senescent p16^Ink4a^ -overexpressing cells could be an efficient strategy to kill RAS+GSE MEFs. Two of the most effective compounds with ‘senolytic’ properties (i.e. selectively toxic against senescent cells) are ABT-263 and ABT-737, well-known anti-cancer agents inhibiting the BCL2 family of anti-apoptotic proteins [10, 11]. RAS+GSE MEFs showed transcriptional induction of two members of the bcl2 family, bcl-2 and bcl-xl, but not of bcl-w (Figure 1F). However, neither treatment with ABT-263 (Figure 1G) nor with ABT-737 (Figure 1H) was toxic for RAS+GSE MEFs, despite the compounds eliminating a significant percentage of MEFs induced to senescence by ionizing radiation (Figure 1G-1H). These data suggest that p16^Ink4a^ overexpressing tumor cells are resistant to currently available compounds with specificity against p16^Ink4a+^ cells.

### Generation of sarcoma cells carrying a p16^Ink4a^-driven suicide gene

**Figure 2:**
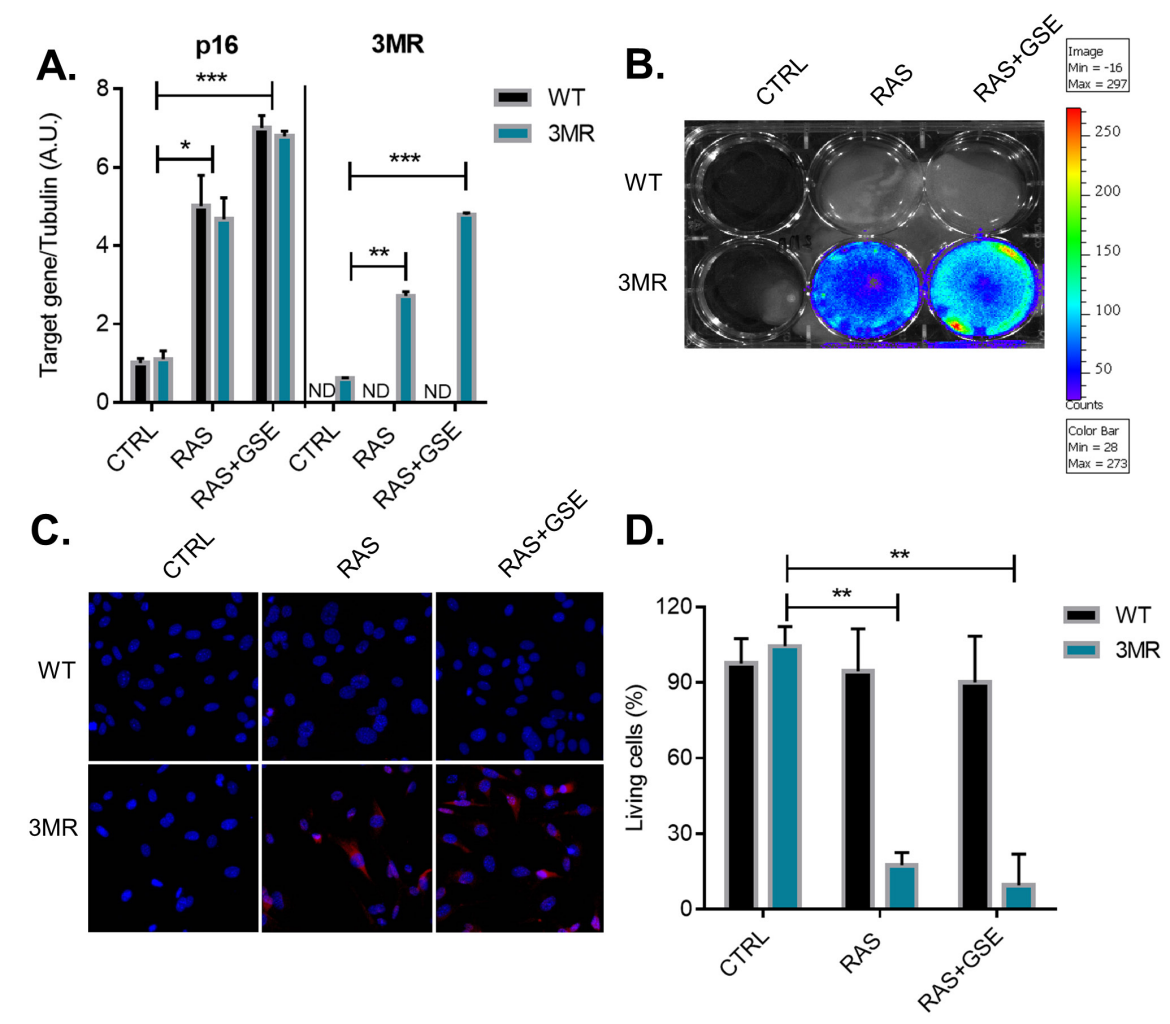
Generation of p16^ink4a^-overexpressing sarcomas carrying the suicide gene p16-3MR Primary Mouse Embryonic Fibroblasts (MEFs) derived from wild-type (WT) or p16-3MR (3MR) mice were transduced with lentivirus containing vector control (CTRL), RASVal12 (RAS) or RASVal12 and p53-GSE (RAS+GSE). **A.** Quantitative real-time PCR (qRT-PCR) analysis of RNA isolated from cells with indicated genotypes. RNA was analyzed for mRNAs encoding p16 and mRFP (as a surrogate for 3MR) relative to tubulin (to control for cDNA quantity). *N* = 3 independent experiments. A.U.=arbitrary units. **B.** Cells were incubated with coelentarazine (substrate for Renilla Luciferase) and luminescence imaged using a Xenogen machine. **C.** Cells were stained with an antibody against mRFP and counterstained with DAPI. **D.** Cells were treated with GCV (10 µg/ml) for 6 days and viability evaluated using a MTS assay. The graph reports the absorbance of each sample as a percentage of control. *N* = 3 independent experiments with 3 technical replicates. *p<=0.05, **p<=0.01, ***p<=0.001.

We then reasoned that an alternative strategy for elimination of p16^Ink4a^ -overexpressing tumor cells could make use of gene targeting therapy. Recently, we have developed a mouse model, called p16-3MR, where cells contain functional domains of Renilla luciferase (LUC), monomeric red fluorescent protein (mRFP), and a truncated herpes simplex virus (HSV)-1 thymidine kinase (tTK) under control of the full *p16*^*Ink4a*^ promoter [12]. LUC and mRFP allow for visualization, and HSV-tTK converts the pro-drug ganciclovir (GCV) into a toxic guanosine analogue, thus allowing for specific elimination of p16^Ink4a^ cells. Similarly to the strategy followed for the generation of wild-type sarcoma cells presented in Figure 1, we derived MEFs from p16-3MR (from now on simply called 3MR) and subjected these cells to RAS overexpression and p53 inactivation (RAS+GSE). Cells overexpressing RAS, but not carrying the p53-GSE modification, were used as control for p16^Ink4a^ activation. The transcriptional induction of the 3MR transgene well correlated with p16^Ink4a^ upregulation in RAS and RAS+GSE cells, suggesting that transcriptional regulatory elements in the promoter of the 3MR transgene were reflecting the endogenous regulation of p16^Ink4a^ (Figure 2A). RAS and RAS+GSE 3MR cells could be identified by luminescence (Figure 2B) and by fluorescence (Figure 2C) which are dependent on the expression of the 3MR components Renilla luciferase and mRFP, respectively. Accordingly, when we treated 3MR cells with GCV we observed toxicity in both RAS and RAS+GSE cells, but not in control cells or WT counterparts. These data suggest that the activation of the 3MR transgene is dependent on p16^Ink4a^ induction but independent from cell cycle arrest. Thus, gene therapy represents an effective strategy for the elimination of p16^Ink4a^-overexpressing tumor cells.

### p16^Ink4a^-driven suicide gene therapy efficiently eliminates p16^Ink4a^-overexpressing sarcoma cells *in vitro* and *in vivo*

**Figure 3:**
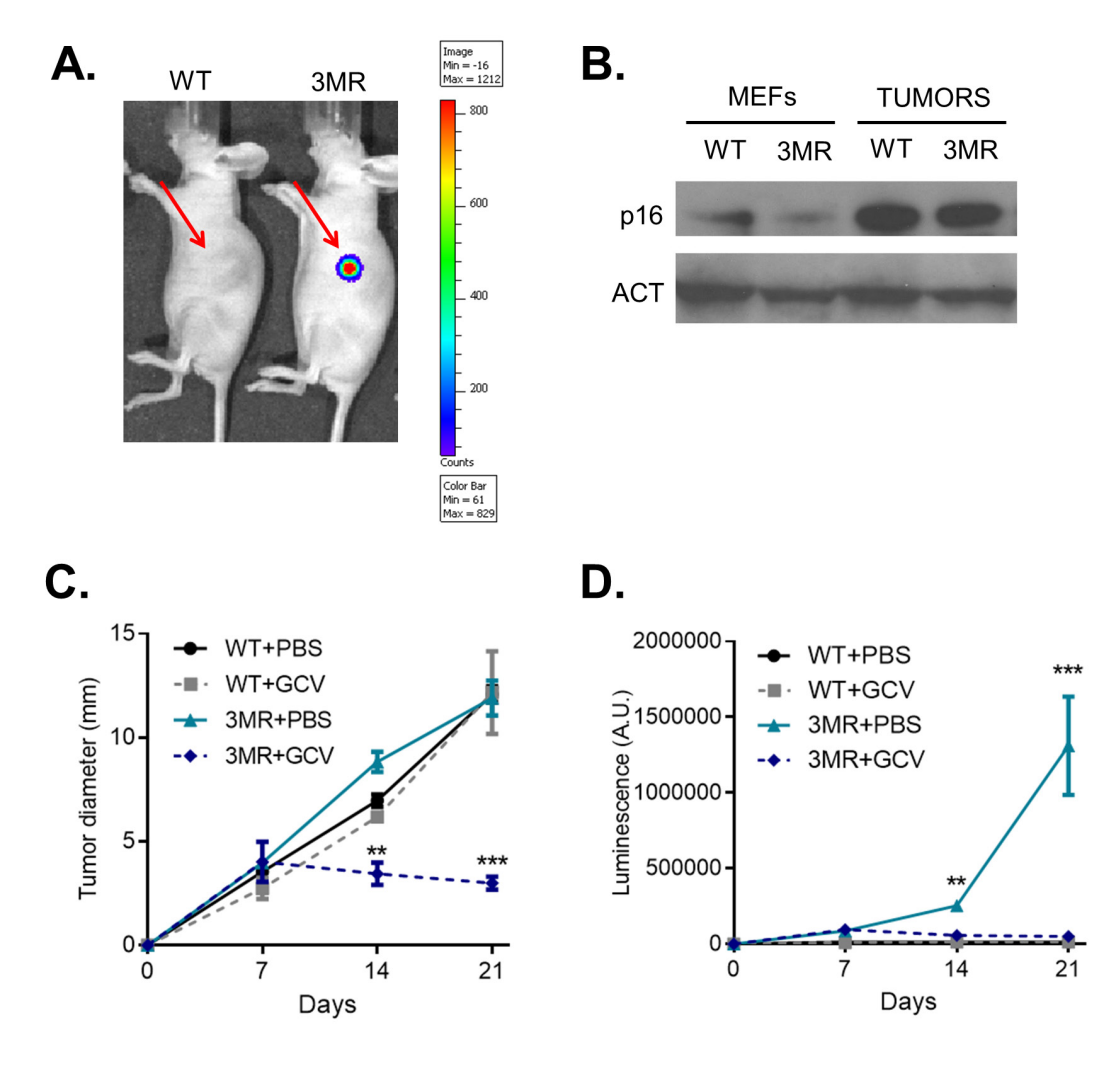
p16^ink4a^-driven suicide gene therapy *in vivo* Wild-type (WT) or p16-3MR (3MR) sarcomas were obtained transducing MEFs with lentivirus containing RASVal12 and p53-GSE (RAS+GSE). 10^6^ cells were sub-cutaneously injected in the flank of nude mice. **A.** 7 days after injection, mice were injected with coelentarazine, and luminescence evaluated using a Xenogen imager. **B.** p16 protein level was measured by immunoblotting using whole cell extracts from RAS+GSE MEFs or tumors. **C.** and **D.** Mice were treated with GCV (daily treatments with 25 mg/kg for 5 consecutive days) or equal voule of PBS at day 7 after cell inoculation. Tumor size was evaluated by using a caliper (C) or by luminescence (D). *N* = 5. *p<=0.05, **p<=0.01.

To study the potential of interfering with p16^Ink4a^-overexpressing cells as anti-cancer therapy *in vivo*, we then inoculated WT or 3MR RAS+GSE cells in immunocompromised mice. 7 days after cell inoculation, we observed tumors of ∼3.5 mm in both groups. Luminescent signal from 3MR cells suggested that p16^Ink4a^ was still induced after inoculation (Figure 3A). To further validate the high level of p16^Ink4a^, we extracted proteins from tumors of both groups, and measured p16^Ink4a^ expression by Western Blotting. Interestingly, p16^Ink4a^ levels were even higher than before cell inoculation, suggesting further induction during proliferation *in vivo* (Figure 3B). Thus, 7 days after inoculation, RAS+GSE 3MR cells showed high level of p16^Ink4a^ and 3MR transgene expression.

We then decided to treat mice with GCV at this stage of tumor progression. Animals inoculated with either WT or 3MR cells were treated daily for 5 consecutive days with GCV (or PBS as control), and tumor growth followed. Strikingly, GCV treatment was sufficient to completely arrest tumor growth in the mice inoculated with 3MR cells, and some signs of regression were also measured (Figure 3C). Accordingly, the luminescence signal was almost completely lost, suggesting successful elimination of 3MR^+^ cells (Figure 3D). We did not observe any toxicity for GCV against WT cells. All together, these data indicate that a killing gene induced by the full *p16*^*Ink4a*^ promoter is an effective strategy to promote tumor regression of p16^Ink4a^-overexpressing sarcomas *in vivo*.

## DISCUSSION

p16^Ink4a^ is an essential player to consolidate the cell cycle arrest of senescent cells. However, in the presence of mutations in key downstream effectors such as RB or CDK4/6, high level of p16^Ink4a^ fails to induce cell cycle arrest. As a consequence, p16^Ink4a^ overexpression is observed in a number of highly aggressive cancer cells [7], and innovative therapeutic approaches could be designed around targeting p16^Ink4a+^ cells. Recently, we and others have identified small molecules that selectively eliminate p16^Ink4a+^ senescent cells [13]. However, when we tested two of these compounds, ABT-263 and ABT-737, well-known anti-cancer agents inhibiting the bcl2 family of anti-apoptotic proteins, we failed in killing p16^Ink4a^-overexpressing murine sarcomas. Possibly, currently available compounds that are selective against p16^Ink4a+^ cells require growth arrest and are not effective against proliferating cells. Moreover, although the elimination of p16^Ink4a+^ cells being a common readout for the screening and validation of senolytics, p16^Ink4a^ is not always up-regulated in all type of senescent cells [14], and thus the toxicity of ABT-263 and ABT-737 (and other senolytics compounds) might not be selective for p16^Ink4a^ overexpression. Suicide gene therapy has been investigated in various types of cancers because of its superior specificity compared to standard genotoxic therapies [15]. A previous effort in testing a suicide gene therapy under the regulation of the *p16*^*Ink4*a^ promoter - the so-called INK-ATTAC system - failed to kill p16^Ink4a+^ cells transformed cells, despite being effective in eliminating p16^Ink4a+^ cells senescent cells [16]. We have recently developed a similar suicide system, called p16-3MR. Major difference is that the p16-3MR gene is under the regulation of the full *p16*^*Ink4*a^ promoter (>50kb), while the INK-ATTAC is regulated by a small portion proximal to the transcription starting site of the INK4a locus (∼2kb). Our strategy, which we have shown being highly effective in non-proliferating cells [12, 17], showed high toxicity for the 3MR sarcomas both in cell culture and *in vivo*. Additionally, since it has been shown that in some instances p16^Ink4a+^ cells are precursor of malignant cells [18], the 3MR system could allow to reduce tumor incidence via removal of p16^Ink4a+^ pre-malignant cells. At this stage, extensive research should to be done to test the toxicity of a p16^Ink4a^ -driven suicide gene therapy strategy against additional tumor types of both murine and human origin, and further characterization of the portion of the *p16*^*Ink4*a^ promoter activated in p16^Ink4a^ -overexpressing cancer cells. Despite recent progress, gene delivery in humans is still not safe and feasible [19]. Moreover, this strategy might target beneficial p16^Ink4a+^ cells, such as the ones associated with wound healing or macrophages [12, 20], and lead to side effects. However, these data together represent a strong proof-of-concept that suicide gene therapy can offer a highly specific intervention for targeting p16^Ink4a^-overexpressing tumors.

## MATERIALS AND METHODS

### Cell preparation and culture

13.5 day embryos were dissected and cultured to produce MEFs, as previously described [12]. All cells were cultured in complete DMEM medium (GIBCO) supplemented with 10% fetal bovine serum and maintained in 3-5% oxygen. Cells were transduced with lentiviruses expressing RASVal12, p53-GSE or control particles, as previously described [21]. For irradiation-induced senescence, cells were exposed to 10 Gy γ-radiation using a ^137^Cesium source and medium was refreshed every 2 days. Cells were exposed to drug treatment at day 10 after irradiation ABT-263 and ABT-737 (Sigma Aldrich) were re-suspended at a stock concentration of 1 mM in DMSO and further diluted in DMEM media for experiment. Cells were treated with 0.313 uM, 0.625 uM, 1.25 uM, 2.5 uM or 5 uM in tripicate wells for each concentration of ABT-263 and ABT-737. Drugs were refreshed every 24 hours for 3 days. On day 4, medium was changed to drug-free medium. On day 5, cell viability was assessed using the MTS assay (Promega) according to the manufacturer’s protocol. For GCV (Sigma-Aldrich), cells were treated with the indicated concentrations for 6 d; the medium was refreshed every 2 d and cell viability was assessed using the MTS assay.

### Mice

p16-3MR and nu/nu mice were maintained in the AALAC-accredited Buck Institute for Research on Aging (Novato, CA, USA) animal facility. All procedures were approved by the Institutional Animal Care and Use Committee. p16-3MR mice were bred in house, and 4 week old nu/nu female mice were purchased from Charles River Laboratories. 10^6^ MEFs were injected sub-cutaneously in the left flank of nu/nu mice. GCV was administered via daily i.p. injections for 5 consecutive days at 25 mg/kg in PBS. Control mice were injected with an equal volume of PBS.

### SA-βgal assay

Cells were plated in a 24-well plate, fixed in a mixture of gluteraldehyde and formaldehyde (2%/2%) for 10-15 minutes and stained overnight with an X-Gal solution using a commercial kit (Biovision). Cells were counter-stained with a 1μg/ml 4′,6-diamidino-2-phenylindole (DAPI, Sigma-Aldrich) solution for 20 min. Images were acquired at 100X magnification, and the number of cells counted by the software ImageJ (https://imagej.nih.gov/ij/).

### EdU staining

Cells were cultured for 24 hours in the presence of EdU, and fixed and stain using a commercial kit (Click-iT EdU Alexa Fluor 488 Imaging kit; Thermo Fisher Scientific). Images were acquired at 400X magnification, quantified using ImageJ (https://imagej.nih.gov/ij/).

### Real time-PCR

Total RNA was prepared using the PureLink Micro-to-Midi total RNA Purification System (Life Technologies). RNA was reverse transcribed into cDNA using a kit (Applied Biosystems). qRT-PCR reactions were performed as described using the Universal Probe Library system (Roche) and primer sets as previously described [12].

### Immunoblot analysis

Cells were washed with warm PBS, lysed, and subjected to SDS-PAGE using 4-12% Bis-Tris gels; separated proteins were transferred to nitrocellulose membranes. Membranes were blocked and incubated for 1 hr at room temperature (RAS: BD Biosciences, #610001; p16: Santa Cruz Biotechnology, #1207; p53, Santa Cruz Biotechnology, #6243; Actin: Cell Signaling, #4970) with primary antibodies. Membranes were washed and incubated with horseradish peroxidase (1:5000; Cell Signaling)-conjugated secondary antibodies for 45 min at room temperature and washed again. Signals were detected by enhanced chemiluminescence.

### Immunofluorescence

Cells on glass coverslips were washed in PBS, fixed in 4% paraformaldehyde, quenched with 50 mM glycine, permeabilized with 0.3% Triton X-100 in PBS, saturated with 3% goat serum (Life Technologies), and incubated with mRFP primary antibody (Allele Biotechnology, #5F8) at room temperature for 1 hour, followed by incubation with Alexa fluorescein-labeled secondary antibodies (Life Technologies) for 45 minutes and mounted using Prolong Fade with Dapi (Life Technologies).

### Bio-luminescence

For *in vitro* luminescence, cells were incubated for 10 minutes with 10 µg of Xenolight RediJect Coelentarazine h (Calipers Life Sciences/Perkin Elmer). Imaging was done using a Xenogen IVIS-200 Optical imaging System. For *in vivo* luminescence, mice were injected i.p. with 15 µg of Xenolight RediJect Coelentarazine h (Calipers Life Sciences/Perkin Elmer). 25 min later, the mice were anesthesized with isofluorane and luminescence measured with a Xenogen IVIS-200 Optical imaging System (Caliper Life Sciences; 5 min medium binning).
